# Steps to achieve carvone-rich spearmint (*Mentha spicata* L.) essential oil: a case study on the use of different distillation methods

**DOI:** 10.3389/fpls.2023.1292224

**Published:** 2023-12-01

**Authors:** Jalil Moradi-Sadr, Mohammad-Taghi Ebadi, Mahdi Ayyari

**Affiliations:** Department of Horticultural Science, Tarbiat Modares University, Tehran, Iran

**Keywords:** spearmint, microwave, hydro-steam, distillation, solvent extraction

## Abstract

**Introduction:**

Spearmint essential oil is a valuable medical and food product. Spearmint essential oil is effective for the treatment of flatulence, indigestion, nausea, and colic along with Alzheimer, obesity, and fungal infections.

**Methods:**

This study evaluated the quality and quantity of spearmint essential oil by examining some extraction strategies. The procedures were hydro-distillation, hydro-steam distillation, microwave-assisted hydro-distillation, and open hydro-distillation. The hydro-distillation had five pH levels (2, 4, 6, 8, and 10) and four NaCl concentrations (0.5, 1, 1.5, and 2%). microwave-assisted hydro-distillation at a power of 225 W was applied for 60, 90, and 120 minutes for process durations. The solvent extraction of herbal distillate obtained by an open hydro-distillation system was done using n-pentane and n-hexane to achieve a recovered essential oil by a rotary evaporator.

**Results and discussion:**

The results showed that the lowest pH in the hydro-distillation process led to obtaining double yield compared to the control. Additionally, at 1 and 1.5% NaCl concentrations, the oil yield increased by 12.86 and 20.87%, respectively. Although the yield was reduced by microwave-assisted hydro-distillation, however within 120 minutes, carvone increased by 12.7% and limonene decreased by 42.3%. The best quality of spearmint oil belonged to solvent extraction followed by rotary evaporator.

## Graphical Abstract

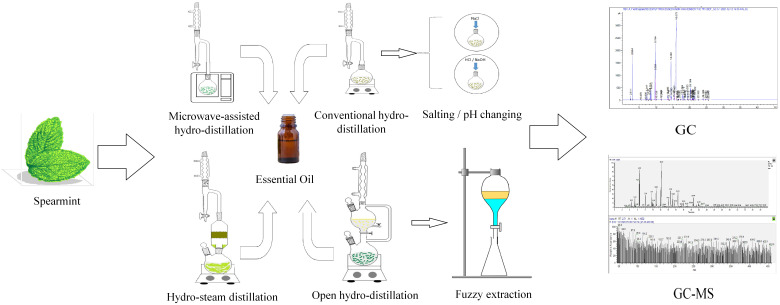

## 1 Highlights

➢ Four methods were used to enrich carvone in the process of extracting spearmint oil.
➢ Hydro, hydro-steam, microwave-assisted and open hydro-distillation were used.
➢ The treatment of maximum acidity of hydro-distillation led to the highest yield.
➢ The yield increased as a result of the salted water used in hydro-distillation.
➢ The microwave treatment has increased carvone and decreased limonene.
➢ The highest quality essential oil produced by open hydro-distillation.

## 2 Introduction

Essential oils (EOs) are secondary metabolic pathway products that have therapeutic and wellness properties (Hussein and El-Anssary, 2019). EO constituents in the proper amount and way of application can numb and ease human disorders (Jamshidi-Kia et al., 2018). EOs have diverse properties such as antifungal (Valente et al., 2023), immune boosting (Sandner et al., 2020), cytotoxicity (Pereira et al., 2017), antioxidant (Nadeem et al., 2022), and antibacterial activities (Wu et al., 2019). Researchers have recently become interested in using Spearmint essential oil (SEO) to preserve foodstuffs, including meat (Shahbazi et al., 2018) and fruit (Yan et al., 2021), rather than occasionally environmentally unfriendly materials.

There are common methods for extracting EOs, including hydro-distillation (HD) (Taraj et al., 2019; Katekar et al., 2022), solvent extraction (Parliment, 2020; Park et al., 2022), steam distillation (Kumar et al., 2022), and modern techniques such as supercritical fluid extraction (Yousefi et al., 2019), microwave-assisted hydro-distillation (MAHD) (Larrazabal-Fuentes et al., 2019; Drinić et al., 2020), solvent-free microwave extraction (Peng et al., 2022; Yingngam et al., 2022), and subcritical water extraction (Cheng et al., 2021). Numerous attempts have been made in recent years to assess the effects of various EO extraction techniques, and it has been discovered that some extraction methods can also result in the loss of specific chemical compounds (Bendahou et al., 2008).

The yield and chemical composition of EOs can be impacted by the various extraction methods and treatments. For example, cold plasma and ultrasound pre-treatments can increase SEOs yield and oxygenated monoterpenes (Moradi-Sadr et al., 2023). Also, it has been verified that MAHD has the potential to improve oxygenated compounds, reduce energy consumption (Drinić et al., 2021), speed up the extraction process, and increase the amount of extracted EO (Yu et al., 2021; Thinh et al., 2022). Supercritical fluid extraction is another interesting procedure for researchers. When compared to other extraction techniques, supercritical fluid extraction can produce the highest yield (Pourmortazavi and Hajimirsadeghi, 2007). This method can selectively extract specific compounds from plant materials and give the EO a unique phytochemical profile (Capuzzo et al., 2013; Uwineza and Waśkiewicz, 2020). Due to the mild extraction conditions, such as low temperature and pressure, this method can preserve the bioactive compounds in EOs (Uwineza and Waśkiewicz, 2020).

In a variety of tropical to temperate climates, *M. spicata* is grown, i.e. Europe, Asia, North Africa, America, India, and Brazil. Its successful cultivation is facilitated by the plains region. In addition to Jammu and Kashmir, Assam, Punjab, Tamil Nadu, Australia, and China are other major distribution areas for *M. spicata* (Li and Tian, 2018). SEO is economically valuable in world marketing, allocating 20% of the globes EO total value in 2019 (Essential Oils (HS: 3301) Product Trade, Exporters and Importers | OEC - The Observatory of Economic Complexity, n.d.). Spearmint is well-known for its therapeutic uses in treating fungal infections, obesity, drug-resistant infections, flatulence, and Alzheimer’s disease (Ali-Shtayeh et al., 2019; Mahboubi, 2021), and also an ingredient agent in dairy farm products, bakeries, mastication gums, toothpaste, and cosmetics (Shaikh et al., 2014). The most important and valuable compound in spearmint is carvone, and spearmint is known as a source of carvone (Morcia et al., 2016), therefore the high proportion of this compound indicates the great quality of SEO. Carvone is responsible for biological activities as well as the distinctive odor of *Mentha spicata* (Mahboubi, 2017), and the three most important SEO ingredients (carvone, limonene, and dihydrocarvone) had insecticidal properties (Yang et al., 2021).

MAHD is a popular technique due to its short process times, increased EO quality and quantity, and lower energy consumption (Moradi et al., 2018; Elyemni et al., 2019; Fardhyanti et al., 2019; Mollaei et al., 2019). MAHD can produce the purest oil with minimally damaged sebaceous glands and a chemical profile similar to HD in hibiscus flower processing (Rassem et al., 2022). MAHD has increased the thymoquinone content in the EO of *Nigella sativa* L. (Abedi et al., 2017). It has caused higher oxygenated compound extraction in *Pogostemon cablin* Benth., (Kusuma and Mahfud, 2017), and *Rosmarinus officinalis* L. EOs (Moradi et al., 2018). MAHD also could extract higher cannabidiol in *Cannabis sativa* L. (Fiorini et al., 2020), and numerous other quality changes in natural product extraction. Researchers have used microwaves for a variety of purposes, i.e., microwave-assisted cloud-point extraction of flavonoids and alkaloids from *crotalaria sessiliflora* L. (Tang et al., 2017), microwave-assisted liquid two-phase extraction for isolation and purification of alkaloids from *Sophora flavescens* Ait. (Zhang et al., 2015), and isolation of genistin, genistein, and apigenin from *Cajanus cajan* L. Millsp. roots using deep mixture solvent-based microwave-assisted extraction (Cui et al., 2015).

It has been proved that the recovered EOs of peppermint and spearmint had significantly higher oxygenated monoterpenes content than the decanted ones. In other words, during hydro-distillation, carvone as the main oxygenated monoterpene slightly dissolves in the water phase (Verma et al., 2011). The amounts of SEO compounds were dedicated as follows by British pharmacopeia (Pharmacopoeia, 2013): carvone (≥55.0%), 1,8-cineole (<2.5%), limonene (2−25%), menthol (<2.0%.), iso-menthone (<1.0%), menthyl acetate (<1.0%.), and pulegone (<0.5%.). Since the high amount of carvone indicates the high quality of the SEO, the open hydro-distillation (OHD) system followed by solvent extraction and the rotary evaporator was used to raise SEO standards.

In this study, the efficacy of various EO isolation methods (three repetitions for each method/treatment) on quantity and chemical composition of SEO was analyzed. The methods included HD (control, HD with diverse pH, and HD salting treatments), hydro-steam distillation (HSD), and OHD system (followed by solvent extraction and rotary evaporator).

## 3 Materials and methods

### 3.1 Plant material preparation

The plant material of *M.spicata* was cultivated at the research farm of the Faculty of Agriculture, Tarbiat Modares University, Tehran, Iran. The plants were harvested in the third year after the planting and naturally shade-dried in the drying room at 25°C for one week. Then the samples were crushed using IKA analytical grinder (A11 IKA) for further analysis.

### 3.2 Essential oil isolation methods

#### 3.2.1 Hydro-distillation procedure

**Figure 1:**
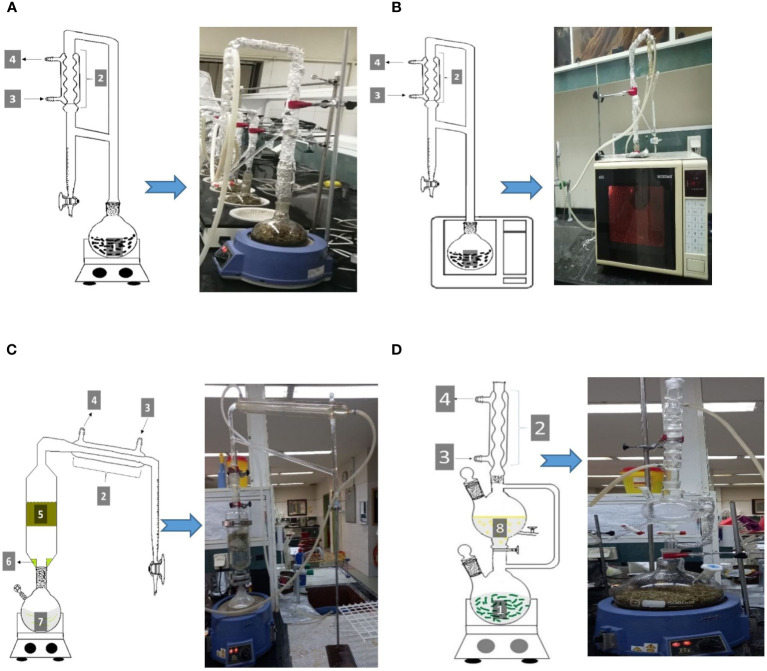
Schematic and graphic illustrations of hydro-distillation **(A)**, microwave-assisted hydro-distillation **(B)**, hydro-steam distillation **(C)**, and open hydro-distillation **(D)** experimental apparatuses; (1: plant material and distilled water, 2: condenser, 3: cold water in, 4: cold water out, 5, plant material container, 6: dropped extract, 7: vapor producer water and returned extract, 8: condensed recovered EO with water).

HD was done using Clevenger-type. A one-liter bottom flask containing 50 grams of the dried spearmint sample was filled with 500 mL of water, as shown in **Figure 1A**, and an electrical heating mantle was used to boil the water. In addition to the control, there were HD treatments that included salted distilled water (0.5, 1, 1.5, and 2% of NaCl), and distilled water with different pH (2, 4, 6, 8, and 10). After three hours of HD, the extracted EO was collected and dried over anhydrous sodium sulfate. Following that, EO was weighed and stored in amber-sealed vials at 4° C for GC and GC-MS analysis.

##### 3.2.1.1 Water pH and NaCl concentration adjustments

The pH of distilled water was adjusted using HCl and NaOH, fitted with a consort C860 pH meter system, and dissolving a specific amount of NaCl to obtain the salty water. In 500 mL of distilled water, salt concentrations of 2.5, 5, 7.5, and 10 grams were dissolved (for concentrations of 0.5, 1, 1.5, and 2%).

#### 3.2.2 Microwave-assisted hydro-distillation procedure

MAHD has been applied using a crafted AEG Micromat 725 E (operating frequency: 50 Hertz and maximum output power of 750 W) operating system (**Figure 1B**). Operational time was chosen as the treatment, and constant power of 30% of the system’s total power (225 W) was fitted. In fact, in 10% of total power, the water was not boiled, while with more than 30% of the total energy, the EO could not be condensed after passing through the condenser and was evaporated from the open side part of the gradual tube of Clevenger apparatus. Fifty grams of the dried sample and 500 mL of water were subjected to the MAHD procedure. The MAHD operated at 60, 90, and 120 minutes and the EO was collected for every stage.

#### 3.2.3 Hydro-steam distillation procedure

The HSD process is shown in **Figure 1C**. Fifty grams of dried sample was used in the HSD procedure and 2500 mL water was utilized to steam production. In the HSD method, the steam passes through the plant material and finally the volatile of plant material along with water were condensed. However, as the steam moves through the plant material, the dry plant sample begins to absorb water. So the plant sample gets heavier over time, the extract drips, and the drops combine with the boiling water in the flask.

#### 3.2.4 Recovered essential oil isolation

A modified distillation apparatus was used to obtain recovered EO (REO) and called the OHD method. In HD, hydrosol circulates in the Clevenger apparatus and separates from EO while in OHD, it is transferred outside to another vessel and through two different valves can be either collected or returned to the distillation flask. The dissolved EO was then separated by a separator funnel using two different solvents, *n*-pentane, and *n*-hexane. **Figure 1D** represents the OHD schematic apparatus.

### 3.3 GC and GC-MS analyses

The composition of all obtained SEO was determined by gas chromatography-mass spectrometry (GC-MS) and the quantity of each component was carried out using an Agilent 7890B gas chromatograph (Agilent Technologies) coupled to a flame ionization detector. An HP-5 capillary column (length 30 m, internal diameter 0.32 mm, and 0.25 μm film thickness) with a temperature program of 2 min at 60°C and then raised to 280°C at the rate of 5°C/min was used in GC. Helium (flow: 1.1 mL/min) was applied in a split ratio of 1:100 as a carrier gas. GC–MS analysis was performed by a Thermoquest–Finnigan gas chromatograph equipped with a fused silica capillary HP-5 column (60 m × 0.25 mm i.e.; film thickness 0.25 μm) coupled with a trace mass spectrometer. The ionization voltage was 70 eV. Ion source and interface temperatures were 200 and 280°C. The mass range was adjusted from 45 to 456 amu. The same oven temperature and carrier gas program were used in GC-MS.

The EO constituents were identified by matching each component’s mass spectra with those of the internal mass spectra library of the main library, Wiley 7.0, and Adams. Further identification was based on a comparison of peak retention indices by using a homologous series of normal alkanes (C8 to C24) verified under the same operating situations and data published in the literature (Adams, 2007).

### 3.4 Statistical analysis

**Figure 2:**
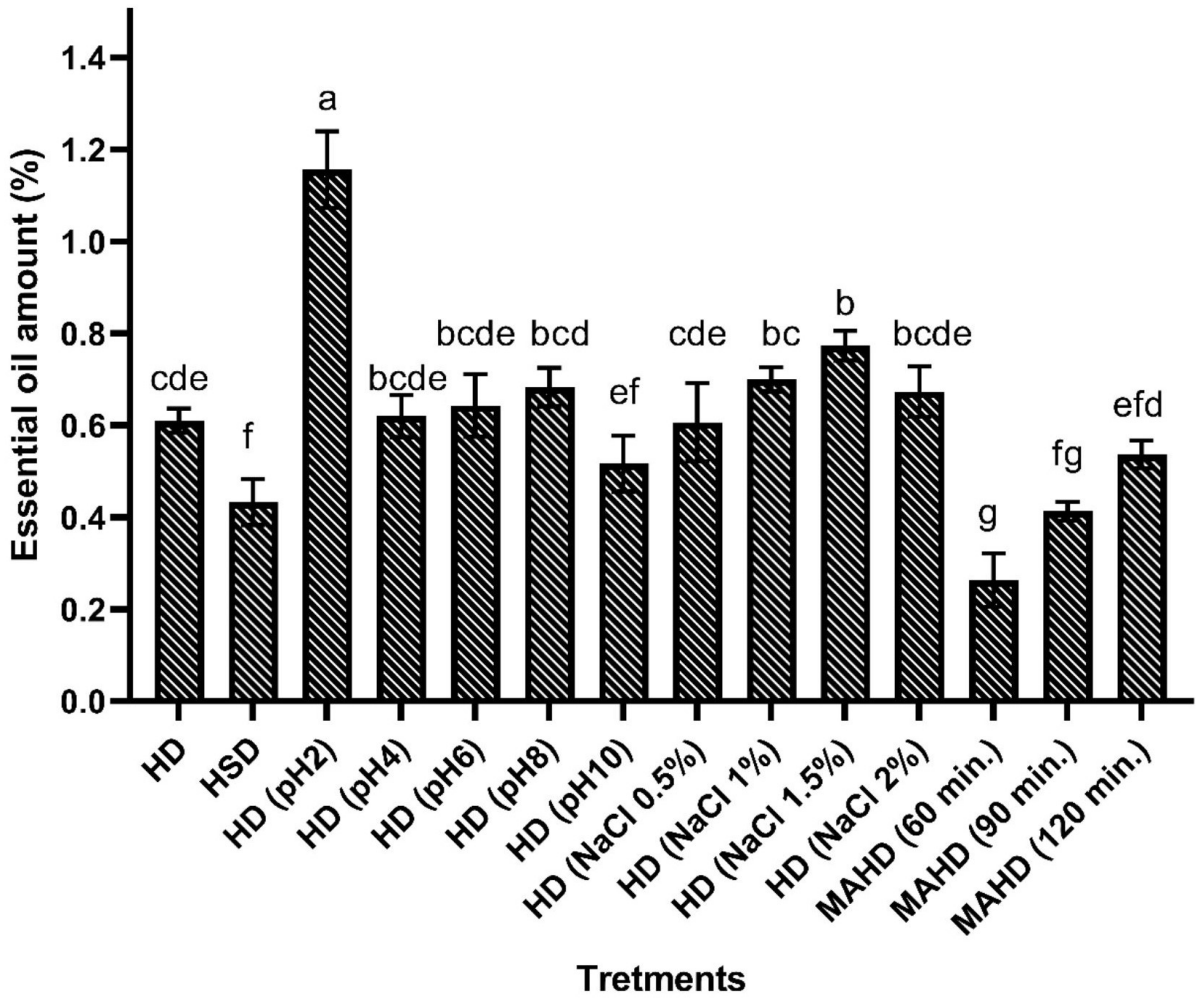
The essential oil content of evaluated procedures. HD: hydro-distillation, HSD: hydro-steam distillation, MAHD: microwave-assisted hydro-distillation.

**Figure 3:**
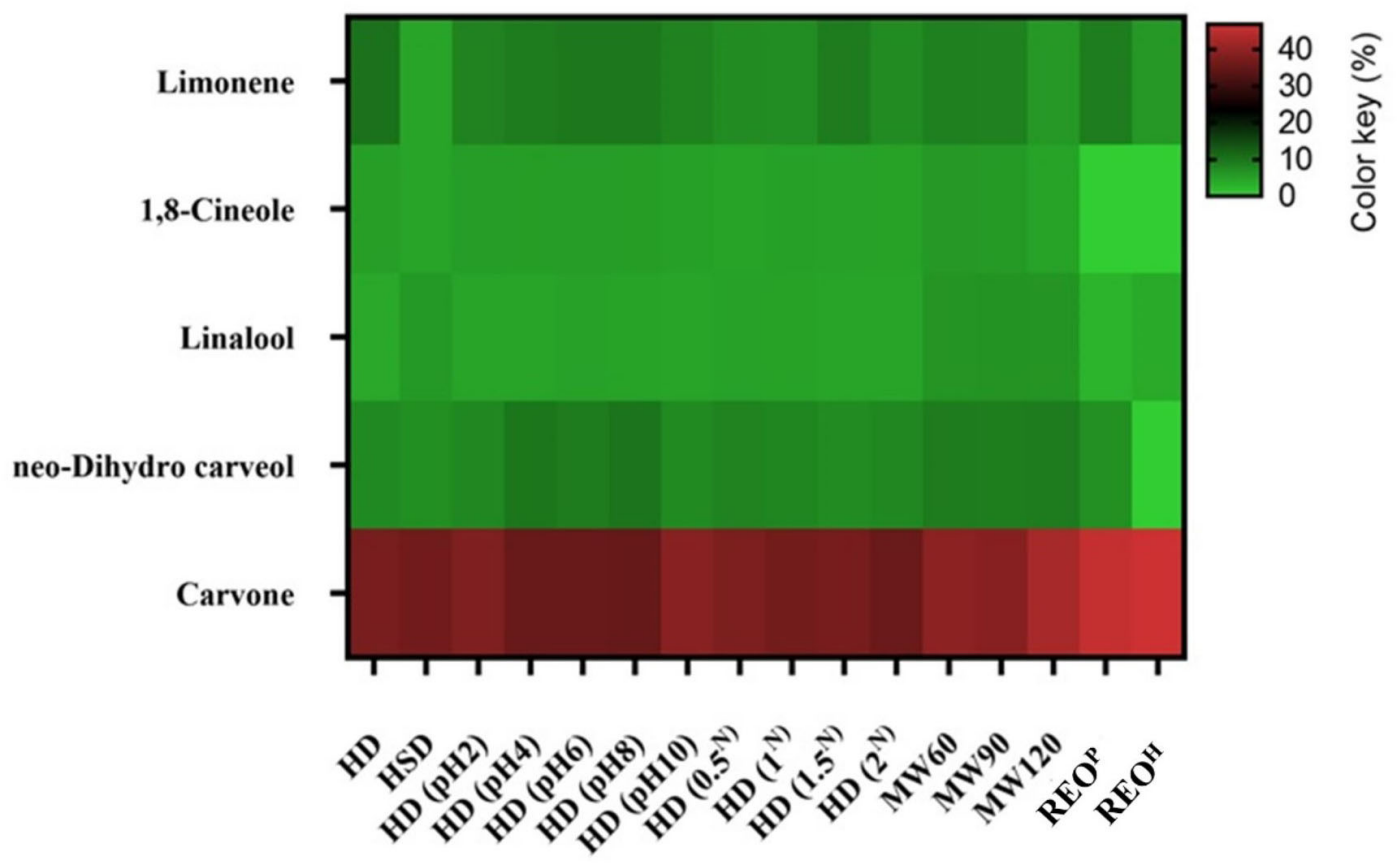
The heat map of major volatile compounds of SEO in control and treatments. HD, hydro-distillation; HSD, hydro-steam distillation; ^N^ percentage of NaCl in salted water; MW, microwave-assisted hydro-distillation; REO^P^, recovered essential oil extracted using n-pentane; and REO^H^, recovered essential oil extracted using n-hexane.

The study utilized a research design known as a completely randomized design. The treatments were assigned randomly to the units. To analyze data, the SAS software system (version 9) was used. Tukeys test at a 5% probability level was used to compare the means of groups. To visualize the results, two figures were created; **Figure 2** represents a yield chart that displays the amount of oil produced by each treatment and procedure, while **Figure 3** illustrates a heat map depicting the percentage of main constituents of SEO in each treatment and method. The figures were drawn with GraphPad Prism 7.0 software.

## 4 Results and discussion

### 4.1 Essential oil yield

The “multiple means” values of the SEOs yield of various treatments are checked using the analysis of variance (ANOVA), as shown in **Table 1**. The treatments’ straightforward effects significantly impacted SEO yield at a 1% probability level. The SEOs yield in several factors ranges from 0.3 to 1.2% (**Figure 2**). The highest and the lowest yields were obtained in 1 hour of MAHD and pH 2 treatment of HD, respectively. The effects of HSD, pH 2, 1.5% of NaCl concentration treatment, and MAHD (processing times of sixty and ninety minutes) (p <0.05) were significant in comparison with the control.

**Table 1:** ANOVA for essential oils yield of different treatment and procedures.

Mean square of the yields (%)	df	Sources
**Treatment**	13	0.13^**^
**Error**	28	0.003
**Coefficients of Variance**	_	8.58

^**^Significance at the 1% probability level, df, degree of freedom.

The SEO yields were found to be 0.61% and 0.43% in HD and HSD methods. In pH treatments, the most quantity of EO was obtained at pH 2 (1.16%), and EO yields of 0.62, 0.64, 0.68, and 0.52% were obtained at pH 4, 6, 8, and 10, respectively. Also in a study on fennel seeds EO extraction, more acidity from pH 6 to 3, led to more yield (Boudraa et al., 2021) which was a similar result to our data. Also in another study, it has been determined that 5% citric acid as a modifier could increase the yield and antioxidant activity of *Rosmarinus officinalis* EO (Dzięcioł, 2021). Lower pHs often destruct cells to remove more compounds and interestingly stimulate the hydrolysis of some glycoside structures to obtain more volatile aglycones (Boudraa et al., 2021). The decrease in yield was also attributed to the hydrolysis of esters as the formation of alcohols and acids that are less volatile compared to the initial esters (Babu and Singh, 2010). When sodium chloride is added to boiling water it can have two effects. Firstly it can raise the boiling point of the water resulting in the extraction of substances that boil at high temperatures. Secondly, it can increase the concentration of the water causing all the substances, in the plant sample to be fully released. This happens because when salt is added to water it breaks down into sodium and chloride ions that interact with the particles of the solvent preventing water molecules from transitioning into the gas state. As a result, the saltwater solution won’t boil at 100°C; instead, it needed to increase the temperature beyond 100°C for it to start boiling (Wu et al., 2020). Also adding salt to distilled water can potentially harm the cells of plant tissue, which in turn makes it easier for water vapor to extract the oil and ultimately increases the yield of essential oil (Suryanti et al., 2023). As realized in previous research, the NaCl concentration of 0.5% did not have a major effect on the EO extraction efficiency (Tunç and Koca, 2019). But in the current research, it was found that the higher concentrations of salt (1 and 1.5%) led to a significant increase in the quantity of EO compared to the control (**Figure 2**). The concentration of NaCl in distillation water had a direct relationship with EO yield, in which the raise of NaCl up to 1.5% led to a high extraction level of EO, however, at 2% the yield was decreased. However, these results contrast with another study in which the NaCl utilization as a modifier for EO extraction of *Coriandrum sativum* L. could not raise the yield (Huzar et al., 2018). Also, the results obtained by (Moradi et al., 2018), showed that the addition of salt just accelerates the extraction process by a salting out phenomenon. In the MAHD process, from 60 to 120 minutes, the EO yield increased (**Figure 2**). However, this result is slightly different from another study in which the amount of EO increased up to 90 minutes during the MAHD process and then decreased with upper time utilization of basil EO extraction (Tran et al., 2018). Also, using MAHD to EO isolation of *Ferulago angulate* has shown that enlarging the time to 40 minutes had a positive effect on the EO yield, and in upper time durations the yield has diminished (Mollaei et al., 2019). It might be due to higher powers applied in their studies which caused the maximum yield obtain earlier. In the upper times of 120 minutes of the MAHD procedure, the EO yield was constant and lower than HD yields.

### 4.2 Essential oils constituents

The results of GC and GC-MS are summarized in **Table 2** and **Figure 3**. The main compounds of the SEO are shown in **Figure 3** as a heat map. Carvone and limonene along with 1,8-cineole, *neo*-dihydro carveol, and linalool are the major compounds in the HD sample with 37, 10.3, 5.1, 7.7, and 4.2%, respectively. Most studies indicated that carvone and limonene are the main components of SEO. Other major constituents were also reported in the literature. For example, germacrene D, piperitenone oxide, and (*E*)-caryophyllene (Farahbakhsh et al., 2021), 1,8-cineole, *cis*-dihydrocarvone, and β-caryophyllene (Bardaweel et al., 2018), or 1,8-cineole, terpinene-4-ol, and β-pinene (Nikšić et al., 2018) are also known as the main constituents of SEO.

**Table 2:** Spearmint essential oils constituents of different isolation treatments and procedures.

No.	Compounds	RI		%															
				HD	HSD	pH2	pH4	pH6	pH8	pH10	0.5^N^	1^N^	1.5^N^	2^N^	MW60	MW90	MW120	REO^P^	REO^H^
**1**	α-Pinene	932		0.1	0.2	0.8	1	1.1	1.1	0.8	0.6	0.6	0.6	0.6	0.5	0.4	0.4	–	–
**2**	Camphene	946		0.2	–	0.2	0.2	0.2	0.2	0.2	0.2	0.2	0.2	0.2	0.1	–	–	–	–
**3**	Sabinene	969		0.6	0.2	0.4	0.5	0.5	0.5	0.5	0.4	0.4	0.4	0.4	0.4	0.4	0.3	–	–
**4**	β-Pinene	974		1.8	0.8	1.5	1.8	1.8	1.9	1.5	1.1	1.2	1.6	1.2	1.2	1.1	0.8	0.6	–
**5**	Myrcene	988		0.6	0.3	0.5	0.5	0.5	0.6	0.5	0.4	0.4	0.5	0.4	0.4	0.4	0.3	–	–
**6**	Limonene	1024		10.3	4.6	8.3	9.2	9.6	9.6	8.5	7.3	7.3	9.2	7.4	8.7	8.6	5.9	9	6
**7**	1,8-Cineole	1026		5.1	4.7	5.4	5.3	5.4	5.3	5	4.7	5.1	4.9	4.8	6	5.7	4.7	–	–
**8**	γ-Terpinene	1054		0.1	–	–	–	–	–	–	–	–	–	–	0.1	0.1	–	–	–
**9**	Terpinolene	1086		0.2	0.3	–	–	–	–	–	–	–	–	–	–	–	–	–	–
**10**	Linalool	1095		4.2	5.9	4.6	4.6	5	4.8	4.6	4.9	4.9	4.5	4.5	6.5	6.6	6.4	2.6	3.9
**11**	1,3,8-ρ-Menthatriene	1108		–	–	0.2	0.2	–	–	–	–	–	–	–	–	–	–	–	–
**12**	3-Octanol acetate	1120		0.2	–	–	–	0.2	0.2	0.2	0.2	–	0.2	–	–	0.2	–	–	–
**13**	Menthone	1148		0.8	0.9	0.9	0.9	0.9	0.9	0.9	0.8	1.4	0.8	1.4	1.9	1.8	0.9	–	–
**14**	*iso*-Menthone	1158		3.6	3.4	3.7	3.3	3.3	3.3	3.9	3.8	3.5	3.4	5	4.6	4.3	4	2.1	3
**15**	Menthofuran	1159		1.7	1.6	1.5	1.4	1.4	1.4	1.5	1.5	3.5	1.5	3.5	–	–	1.6	1.4	1.2
**16**	Borneol	1165		0.5	0.5	–	0.6	–	–	–	–	0.8	0.5	0.8	0.9	–	0.8	1	0.8
**17**	Terpinene-4-ol	1174		–	–	0.7	–	0.6	0.6	0.6	0.5	0.6	0.5	0.6	0.7	0.7	–	–	–
**18**	*neo*-Dihydro carveol	1193		7.7	7	7.8	9.8	9.2	10	7.6	8.4	8	7.5	7.8	9.2	9	9.3	6.9	–
**19**	*cis*-4-Caranone	1200		0.2	0.6	0.3	0.4	0.3	0.3	0.3	0.4	0.3	0.3	0.3	0.5	0.5	0.5	2	4.9
**20**	α-Terpineol	1205		–	–	–	–	–	–	–	–	–	–	–	–	–	–	–	–
**21**	*cis*-Carveol	1226		0.6	0.6	0.8	0.7	0.7	0.8	–	0.5	0.5	0.5	0.6	0.4	0.5	0.5	–	2.1
**22**	Pulegone	1233		2.4	2.4	2.9	2.7	2.7	2.9	2.9	3.1	3.5	3.3	3.4	3.1	3.3	3	–	–
**23**	Carvone	1239		37	36.2	37.9	35.2	35.1	34.8	38.9	37.4	36.3	36.8	35.3	39.3	38.7	42.3	45.7	46.8
**24**	Piperitone	1249		0.4	0.4	0.6	0.5	0.5	0.5	0.6	0.5	0.5	0.4	0.5	0.4	0.5	0.5	0.3	0.3
**25**	*neo*-Isopulegyl acetate	1274		0.1	–	–	–	–	–	–	–	–	–	–	–	–	–	–	0.2
**26**	Isobornyl acetate	1283		0.2	0.4	0.3	0.2	–	–	0.3	0.4	0.3	0.2	–	0.1	0.1	0.3	0.3	0.2
**27**	*neo*-Dihydro carveol acetate	1306		0.2	0.3	0.2	–	–	–	0.2	0.2	–	0.2	–	0.2	0.2	0.2	0.3	0.3
**28**	*iso*-Dihydro carveol acetate	1326		1.6	2.3	1.6	1.4	1.5	1.4	1.7	1.6	1.5	1.4	1.5	1.8	1.8	1.9	3	2.4
**29**	Piperitenone	1340		4	2.4	4.4	5	5	4.7	4.1	3.7	4.3	4.5	4.5	2.6	2.9	3.3	3.9	4.3
**30**	*cis*-Carvyl acetate	1365		0.5	0.6	0.5	0.5	0.5	0.5	0.5	0.5	0.5	0.5	0.5	0.5	0.5	0.5	0.7	0.7
**31**	Isoledene	1374		1	2.5	1.1	1	1.1	1.1	1.1	1.1	1.1	1.1	1	0.2	0.9	1	1.7	3.5
**32**	β-Bourbonene	1387		3.4	1.9	3.6	4.1	4.1	3.9	3.2	3	3.5	3.8	3.6	2.1	2.4	2.6	2.8	1.7
**33**	β-Elemene	1389		–	–	–	–	–	–	–	–	–	–	–	0.8	–	–	–	–
**34**	Longifolene	1407		–	–	–	0.2	0.2	0.3	–	–	–	–	–	0	–	–	–	–
**35**	α-Thujaplicin	1410		0.2	0.2	0.2	0.2	0.2	0.2	0.2	–	–	0.3	0.2	0.2	0.2	–	0.3	0.3
**36**	(*E*)-Caryophyllene	1417		3	7.2	3.1	3	3.1	3.1	3.2	3.2	3	2.4	2.8	2.4	2.8	2.9	2.9	5
**37**	β-Gurjunene	1431		0.2	–	–	–	–	–	–	–	–	–	–	–	–	–	–	–
**38**	α-Humulene	1452		0.2	–	0.3	0.2	0.3	0.3	0.3	0.4	0.2	0.2	0.3	0.2	0.2	0.3	–	–
**39**	*trans*-Cadina-1(6),4-diene	1475		0.9	2.1	0.8	0.8	0.9	0.9	0.9	0.8	0.8	0.7	0.7	0.7	0.8	0.8	0.2	0.9
**40**	γ-Muurolene	1478		0.2	0.4	–	0.2	–	–	–	–	0.3	–	–	–	0.2	–	0.2	0.3
**41**	γ-Himachalene	1481		0.3	0.5	0.2	0.3	0.2	0.2	0.2	0.4	0.3	0.4	–	–	0.3	0.2	–	–
**42**	D-Germacrene	1484		–	–	–	–	–	–	–	–	–	–	–	0.2	–	0.2	–	–
**43**	*trans*-Muurola-4(14),5-diene	1493		–	–	0.2	–	0.2	0.2	0.3	0.2	–	–	0.3	0.3	–	–	0.3	0.3
**44**	Bicyclogermacrene	1500		0.2	0.7	–	–	–	–	–	–	–	–	–	–	0.2	–	–	–
**45**	γ-Cadinene	1513		–	0.6	–	–	–	–	–	–	–	–	–	–	–	–	0.4	0.6
**46**	*cis*-Calamenene	1528		1.2	0.5	0.3	0.2	0.2	0.2	0.3	0.3	–	0.2	0.2	0.2	0.2	0.2	0.5	0.5
**47**	Spathulenol	1577		1.4	2.8	1.8	1.7	1.7	1.6	1.8	2.2	1.9	2	2	1.4	1.3	1.6	2.1	1.7
**48**	β-Atlantol	1608		0.3	0.6	0.4	0.4	0.4	0.4	0.4	0.5	0.4	0.5	0.5	0.3	0.3	0.3	–	–
**49**	Torreyol	1644		0.2	0.4	0.9	–	0.3	–	–	–	–	–	–	–	–	–	0.4	0.4
	Total			98.3	96.9	98.9	98.4	98.6	98.4	97.7	95	96.95	95.94	97	95.9	96.5	98.8	97.9	98.8

^N^NaCl concentration (%), MW(n): MAHD with (n) minutes of processing time, REO^P^: recovered EO extracted by n-pentane, REO^H^: recovered EO extracted by n-hexan.

**Figure 4:**
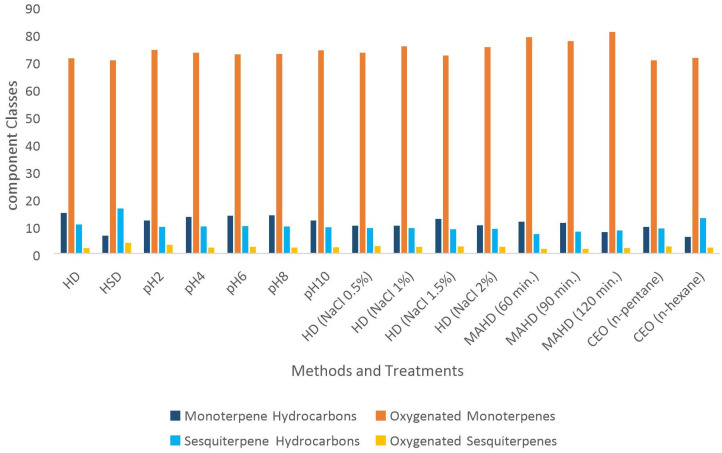
Classification of the SEOs components. HD, hydro-distillation; HSD, hydro-steam distillation; MAHD, microwave-assisted hydro-distillation; REO, recovered essential oil.

However, oxygenated monoterpenes especially carvone and limonene, have been reported as the main components in SEO in different studies (Ali-Shtayeh et al., 2019; Mokhtarikhah et al., 2022). The oxygenated monoterpenes quantity was considerably higher in REO extracted with *n*-pentane and *n*-hexane in comparison with the control. Also, there was a minimum amount of extracted oxygenated monoterpenes in typical HD (**Figure 4**).

#### 4.2.1 Essential oils composition of hydro-distillation and hydro-steam distillation

In HD and HSD EOs, 42 compounds were identified. As shown in **Figure 4**, oxygenated monoterpenes were the major class of chemical profile in all of the EOs. Monoterpene hydrocarbons were also the second major chemical group of EO in HD. However, in the case of HSD, the second major chemical group was the sesquiterpene hydrocarbons. There was no significant difference in the carvone content of HD and HSD EOs. Despite that, the limonene content was decreased by 55.4% in HSD, and also the HSD procedure isolated the higher amount of linalool (**Table 2**). The quantity of extracted sesquiterpenes in the HSD process was also higher than the control. These results were similar to the conclusion reached in an article in which the HSD method increased the amount of monoterpene and sesquiterpene hydrocarbons in *Carum carvi*, *Anethum graveolens*, and *Pimpinella anisum* EOs (Mohammed et al., 2019).

#### 4.2.2 Phytochemical effect of hydro-distillation water pH on oil composition

The limonene content of pH-treated resultant EOs was lower than the control and in pH 2 and pH 10 was slightly lower than other pH levels. However, the proportion of *iso*-menthone was slightly higher at pH 2 and pH 10. Also as shown in **Table 2**, carvone, the main component of SEO, in minimum and maximum pHs, was raised compared with other pHs and control. The amount of *neo*-dihydro carveol was enhanced by increasing pH to 8 and then reduced to pH 10.

#### 4.2.3 Effect of modified hydro-distillation using different NaCl concentrations on essential oil profile

**Table 2** represented the effect of 0.5, 1, 1.5, and 2% of NaCl on the SEO profile. In all four treatments, limonene content did not reach the control samples although 1.5% of NaCl had the most limonene amount compared to other treatments. The content of linalool was increased in all concentrations of NaCl. The highest *neo*-dihydro carveol content was obtained in 0.5% treatment as compared with other concentrations and control. Also, this treatment led to obtaining higher carvone content. The treatment of 2% raised the *iso*-menthone content. The piperitenone percentage was also upper in 1, 1.5, and 2% treatments. The impact of NaCl on EO composition has also been mentioned in another study in which the camphor of *Coriandrum sativum* L. EO was raised using NaCl in distilled water (Huzar et al., 2018).

#### 4.2.4 Qualitative efficacy of microwave-assisted hydro-distillation on oil composition

The MAHD procedure also affected the SEO quality. The carvone content was enhanced by MAHD and also the highest amount of the component was found in 120 minutes duration. The limonene percentage was 8.7, 8.6, and 5.9 in 60, 90, and 120 minutes of the MAHD, respectively, while the percentage of limonene was 10.3 in control. MAHD used by another research led to a different result in which the content of carvone in EO of *M. piperita* L. has decreased, however, limonene percent was improved (Abdel-Hameed et al., 2018). The piperitenone was also diminished in this method. The amounts of *neo*-dihydro carveol, linalool, and *iso*-menthone were upper in MAHD, and *iso*-menthone had an inverse correlation with MAHD interval (**Table 2**).

#### 4.2.5 The recovered essential oil constituents

The REO was extracted by the exploitation of two different solvents to achieve better results. The amount of both main compounds (carvone and limonene) in REOs extracted by *n*-pentane and *n*-hexane showed that REOs contain more of these two compounds. Also as shown in **Table 2**, there was no noticeable distinction in carvone proportion in REOs. However, the content of carvone in both samples was the utmost compared to all of the studied treatments and control. The carvone and limonene contents (45.7 and 9%) extracted by *n*-pentane, and also the amounts in the *n*-hexane sample (46.8 and 6%) revealed that the quality was improved using solvent extraction. The resultant chemical profile indicated that carvone is partially miscible with hydrosol, which was also previously proved (Çam et al., 2018). The key distinction within the solvents extraction method was the extracted contents of *neo*-dihydro carveol by *n*-pentane which was considerably higher than in the *n*-hexane.

This phenomenon established that hydrosols contain a decent amount of carvone and limonene which can be extracted by solvent to have the highest quality of SEO. However, hydrosol itself is usually consumed as a carminative in many places around the world because of its carvone content.

## 5 Conclusion

The results showed that the acidification of distilled water had some advantages on the quality and quantity of EOs. The highest acidity (pH 2), raised the EO yield about 2 times and increased the oxygenated monoterpenes compared to the HD as control treatment. In other words, the SEO yield was multiplied, and additionally, the quality of SEO which is related to carvone concentration, was higher than the control in the highest acidity treatment. Enhancements of 12.86 and 20.87% in EO yield were also obtained at 1 and 1.5% of salt concentrations, respectively, with a similar quality to the control. All 3 MAHD treatments led to less EO extraction than the control. However, more processing time improved the SEO content (in comparison with lower times). In 120-minute MAHD, the quantity of carvone increased by 12.7% compared to the control, but the amount of limonene was reduced by 42.3%. The OHD method followed by liquid-liquid partitioning with *n*-pentane and *n*-hexane solvents, not only increased the yield of EO content but also could isolate more carvone, which is the main carminative structure and the characteristic marker in the quality of SEO. Nevertheless, the HSD technique reduced the SEO yield by 32.8% compared to the control. By the way, HSD could increase sesquiterpene hydrocarbon content.

## Data availability statement

The original contributions presented in the study are included in the article/Supplementary Material. Further inquiries can be directed to the corresponding author.

## Author contributions

JM-S: Data curation, Formal Analysis, Investigation, Methodology, Software, Validation, Visualization, Writing – original draft. M-TE: Formal Analysis, Funding acquisition, Project administration, Resources, Supervision, Validation, Writing – review & editing. MA: Investigation, Methodology, Writing – review & editing.
